# Editors’ pick: re-‘colon’-ization of healthy microbiota after recurrent C. difficile infection

**DOI:** 10.1186/2041-2223-4-28

**Published:** 2013-12-16

**Authors:** Bruce Budowle

**Affiliations:** 1 Institute of Applied Genetics, Department of Forensic and Investigative Genetics, 3500 Camp Bowie Blvd University of North Texas Health Science Center, Ft Worth, TX 76107, USA; 2 Center of Excellence in Genomic Medicine Research (CEGMR), King Abdulaziz University, Jeddah, Saudi Arabia

## 

The story of Kaitlin Hunter [1] brings to light the potential triumphs of the age of biotechnology and how an understanding of the interplay of genomics and the human microbiome can provide a cure to a life-threatening disease. After a serious car accident and under standard healthcare, Kaitlin was given antibiotics to prevent infection. Likely, the antibiotic treatment compromised her normal gastrointestinal flora and permitted *Clostridium difficile*, a toxin-producing, Gram-positive, anaerobic, spore-forming bacillus, to become established. *C. difficile* causes diarrhea and is linked to approximately 14,000 deaths each year in the United States alone [2]. However, a novel solution was undertaken to treat Kaitlin - her mother, a healthy donor, provided a fecal sample which was transplanted into Kaitlin’s colon. Her mother’s bacteria re-colonized the colon and Kaitlin was cured. This life-saving treatment may make one reconsider the phrase of ‘taking no “_ _ _ _” from anyone’.

While a fecal transplant makes logical sense for staving off a *C. difficile* infection, there are patients (and physicians) who may not opt for such a treatment. First, patients may not relish the thought of receiving fecal matter that would be infused directly into their colons. The thought of it is unpleasant at best. Second, a fecal sample even from a healthy donor would be replete with bacteria, some of which may be pathogenic or at least opportunistic pathogens. However, Petrof et al. [3] have devised a solution to ‘RePOOPulate’ the intestines of patients with *C. difficile* infections. They used investigative genetics technology to develop a stool substitute, which poses less risk to the patient, and probably for the first time employed massively parallel sequencing (MPS) to track a transplant and monitor a patient’s recovery. Thereby, they demonstrated that the disruptive MPS technology has immediate diagnostic applications.

Petrof et al. obtained a stool sample from a healthy, 41-year-old woman and isolated 62 different bacteria. After rejecting antibiotic-resistant microbes - those that may cause disease, 33 intestinal bacteria isolates remained. These isolates were reconstituted in proportions to approximate those from the original stool sample. Thus Petrof at el. created a stool substitute mixture comprised of a multispecies community no longer in the presence of fecal material.

Two patients were selected for ‘repoopulation’. They were a 74-year-old Caucasian woman with six episodes of recurrent *C. difficile* infection over an 18-month period and a 70-year-old Caucasian woman with a history of peripheral neuropathy, which predisposed her to recurrent skin and soft tissue infections and under cefazolin treatment developed a *C. difficile* infection. Both were infected with a particularly hypervirulent strain of *C. difficile*, ribotype 078. These patients posed a challenge because of age and recurrent infection.

The status of pretreatment and post-treatment microbiota was monitored by sequencing PCR products of the bacterial V6 rRNA region on the Ion Torrent platform with low to medium throughput chips for the instrument. The Ion Torrent is one of the commercially available massively parallel sequencers that enables genomic analyses on the bench top of most laboratories which was only a short time ago the domain of large genomic centers. Throughput of the sequencing chips is upwards of 100 megabases and for single patient monitoring was deemed sufficient to obtain a depth of coverage to assess diversity of the patients’ intestinal microbial flora. Up to 12 samples were multiplexed on each chip through use of indexing. Therefore community microbial composition analysis could be assessed in virtually real-time (that is, 24 to 48 h) to track the success or failure of the stool substitute transplant to re-establish the microbiota of the patients.

Sufficient V6 rRNA reads were obtained (between 3,758 and 76,752 V6 rRNA reads per sample for Patient 1 and between 19,751 and 64,200 reads per sample for Patient 2) to estimate general and relative diversity of the microflora of the patients. Within 2 to 3 days of stool substitute treatment, each patient recovered a normal bowel pattern and remained symptom-free for up to 6 months when monitoring was stopped. The first patient displayed a highly diverse microbiota that after treatment became less diverse and after 6 months regained similar diversity as that of pretreatment. The second patient’s microbiota had relatively low diversity and became more diverse following treatment which stabilized with a higher diversity than that of pretreatment. Genomic analyses demonstrated that the rRNA sequences representative of the stool substitute were infrequent in the pretreatment stool samples of the patients. Once stabilized, the same sequences comprised >25% of the patients’ samples.

Taxonomic assignment with rRNA V6 region could only be carried out at the family level as greater phylogenetic resolution cannot be attained using a single house-keeping gene. This lack of resolution did not compromise monitoring for stool substitute transplantation success. However, given the throughput of MPS, adding a few more housekeeping genes could easily provide species/strain level resolution and even monitor for mutation that may arise.

Although successful, the RePOOPulation study was limited to two patients and more studies are needed. However, Petrof et al. have demonstrated the proof of concept that this stool substitute, synthetically reconstituted from fecal material, can be an effective treatment for *C. difficile* infection, particularly for recurrent infections with hypervirulent strains. The authors point out that there are several advantages to using a substitute over actual fecal material, besides the obvious distaste of having such material injected into one’s body. These include ‘the exact composition of bacteria administered is known and can be controlled; the bacterial species composition can be reproduced, should a future treatment be necessary; preparations of pure culture are more stable than stool, which some groups recommend should be collected fresh and instilled into the recipient within 6 h of collection; an absence of viruses and other pathogens in the administered mixture can be ensured, thereby improving patient safety; and the administered organisms can be selected based on their sensitivity to antimicrobials, allowing an enhanced safety profile’.

It is hard to believe that only a few years ago the human microbiome was defined solely by *E. coli* and bacteria that caused bad breath and tooth decay. The diversity of the human microbiome is substantial and its secrets, as they unravel, promise to bring additional insights to human health and potential treatments to improve well-being. Investigative genetics tools, such as high throughput sequencing and targeted gene diagnostics, will figure prominently into our personalized medical treatment and fecal, or simulate, transplantation is just the tip of the iceberg. Who knows… the next time one of us travels to an area of the world where the microflora of the water can cause intestinal discomfort, a possible pretreatment for better well-being during travel may be a stool simulant enema.

## Competing interests

This author has no competing interests.
